# Modelling the relative contribution of infection, routine vaccination and supplementary immunisation activities to measles seroconversion in Kenyan Children

**DOI:** 10.1371/journal.pcbi.1013531

**Published:** 2025-09-22

**Authors:** Caroline Mburu, John Ojal, Rose Selim, Rose Ombati, Donald Akech, Boniface Karia, James Tuju, Antipa Sigilai, Gaby Smits, Pieter van Gageldonk, Fiona van der Klis, Eunice Kagucia, Anthony Scott, Ifedayo Adetifa, Stefan Flasche

**Affiliations:** 1 KEMRI-Wellcome Trust Research Programme, Kilifi, Kenya; 2 Department of Infectious Diseases Epidemiology, London School of Hygiene and Tropical Medicine, London, United Kingdom; 3 Department of Immunosurveillance, Centre for Infectious Diseases Control, National Institute of Public Health and the Environment (RIVM), The Netherlands; 4 Charite Centre for global Health, Charite-Universitaetsmedizin, Berlin, Germany; Stockholms Universitet, SWEDEN

## Abstract

**Background:**

Measles outbreaks continue to cause a large burden of disease in Africa including Kenya. We used information from regular serological surveys in Kilifi Health and Demographic Surveillance System (KHDSS) in combination with mathematical modelling to estimate the relative contribution of the vaccination programme to current measles immunity.

**Methods:**

We developed a static birth cohort model to track the proportion of children who are either measles naïve or seroconverted due to natural infection or vaccination through first dose of measles-containing vaccine (MCV1), the second dose (MCV2), or supplementary immunisation activities (SIAs). We fitted the model to biennial paediatric serological survey and case notification data and used vaccination coverage estimates from the KHDSS to estimate the relative contributions of vaccination and infection to measles immunity in Kilifi.

**Results:**

We estimated that between 2009 and 2021, 60% (95%CI 55–64%) of measles seroconversion in Kilifi was attributable to MCV1, with MCV2 contributing 1.0% (95%CI 0.9-1.1%) since its introduction. Natural infection and SIAs accounted for 24% (95%CI 17–31%) and 16% (95%CI 14–19%), respectively. A hypothetical 10% increase in MCV1 coverage increased the seroconversion attributed to MCV1 to 67% (95%CI 63–71%), with concurrent reductions in seroconversion from natural infection and SIAs to 13% (95%CI 9–18%) and 10% (95%CI 9–12%), respectively. Importantly, this same 10% increase in MCV1, if administered promptly at 9 months, could potentially reduce seroconversion from natural infection further from 24% to 11% (95%CI 07–15%) and reliance on SIAs from 16% to 8% (95% CI 7–10%).

**Conclusion:**

Optimizing routine coverage timing and uptake is crucial for reducing SIAs dependence and measles susceptibility. A 10% MCV1 coverage increase could have halved susceptibility and lessened SIA demand, highlighting the potential of minor improvements in coverage to alleviate measles and reduce costly SIAs.

## Author summary

Despite a comprehensive vaccination programme of two measles doses and frequent SIAs, outbreaks continue to be experienced in Kenya and the country has not eliminated measles in line with continental and global targets. Here, we developed a static cohort modelling framework to improve our understanding of the observed measles immunity and the relative contribution of routine immunisation (RI), SIAs, and natural infection in shaping the current immunity profile. Using this framework alongside measles case-notification data, records on timing, schedule and coverage of RI and SIAs, and historical serological data spanning over a period of 12 years, we show that both the timing and uptake of the first-dose programme need to be optimised as this contributes the most to seroconversion and has the highest impact on reducing reliance on frequent SIAs and residual susceptibility.

## Background

Measles remains a major cause of morbidity especially in low and middle income countries (LMICs) [1] and one of the major causes of mortality in children younger than 5 years [2]. Measles outbreaks continue to be reported in Africa [3], including Kenya [4,5], and in many high income countries too [6,7]. A decline in immunisation coverage and delays in supplementary immunisation activities (SIAs) during the COVID-19 pandemic have further increased the risk of outbreaks [8,9]. Owing to the very high transmissibility of measles, local elimination requires high population immunity of about 90–95% in a randomly mixing population [10] which is often beyond the coverage that can be achieved by Routine Immunisation (RI). At least 95% coverage for both the 1^st^ dose of measles containing vaccine (MCV1) and the 2^nd^ dose (MCV2) is recommended by WHO for elimination [11]. Thus, regular SIAs are often required to complement the suboptimal coverage achieved by RI. In addition, the WHO recommends continued monitoring via serological surveys to identify immunity gaps [11].

In Kenya, MCV1 was introduced in 1980 and is administered at 9 months of age. Administrative coverage for MCV1 has ranged from a minimum reported national coverage of 60% to a maximum reported coverage of 93% since its introduction up to 2021 [12]. MCV2 was introduced in 2013 and is administered at 18 months albeit uptake has been poor at 28% to 57% [12]. Since 2002, SIAs have been conducted every 3–4 yrs in either children younger than 5 or 15 years old and have typically achieved more than 80% coverage [4]. Nevertheless, measles outbreaks are regularly reported, and Kenya has not eliminated measles.

In Kilifi, where health and demographic surveillance has been running since 2000 [13], regular serological surveys have been conducted since 2009 alongside monitoring for vaccine uptake and measles surveillance. We use this unique data in combination with mathematical modelling, to estimate the relative contribution of the MCV1, MCV2, SIAs and natural infection to age-specific measles immunity profiles. We then use that model to predict how increases in MCV1 and MCV2 coverage may reduce the need for SIAs and the burden of measles infection.

## Methods

### Ethics statement

Ethical approval was obtained from the Scientific and Ethics Review Unit (SERU) of the Kenya Medical Research Institute (Protocol SERU 3847). The serological samples were collected under a SERU-approved protocols with a provision for storage of residual samples and use in future research (SERU 1433,4085,2887,3149,3426) Written informed consent was obtained from parents/legal guardians of all participants prior to sample collection. In addition, written assent was obtained from all participants aged 13–14 years old.

### Serological data

The serology data utilised for model development spanning from 2009 to 2019 originated from two cross-sectional surveys: the Malaria Cross-Sectional Survey [14,15] which is in part longitudinal and the Pneumococcal Conjugate Vaccine Impact Study (PCVIS) [16] which is primarily cross-sectional in nature. These surveys were conducted within the Kilifi Health Demographic Surveillance System (KHDSS) [13] as part of an ongoing effort to actively monitor malaria and pneumonia infections in children under 15 years of age. The data in 2021 was from participants who were recruited from COVID-19 serosurveillance in Kenya conducted as part of the pandemic response [17]. IgG antibodies against measles were determined using a fluorescent bead-based multiplex immunoassay [18]. The comprehensive details on data collection and sample testing procedures have been described elsewhere [19].

### Vaccination coverage

**Fig 1:**
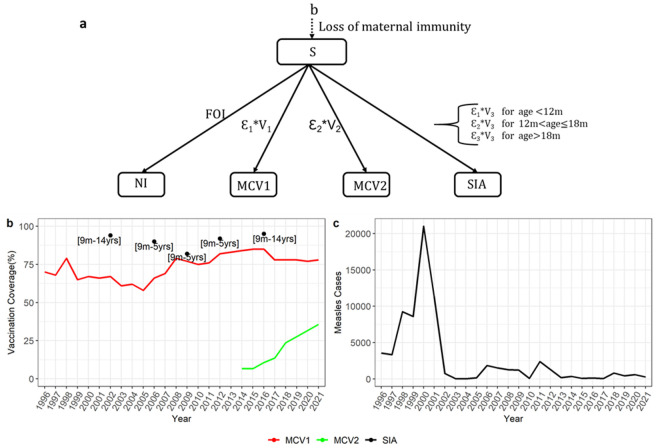
1a shows a static cohort model of measles immunity. Individuals can be divided into 5 mutually exclusive states: S-susceptible, NI = Naturally Infected, MCV1 = Seroconverted from MCV1 dose, MCV2 = Seroconverted from MCV2 dose, SIA = Seroconverted from SIA dose. Ɛ_1_ = 1-vaccine failure of one dose given at less than 1 year, Ɛ_2_ = 1-vaccine failure of one dose given between 12–18 months and Ɛ_3_ = 1-vaccine failure of one dose given at 18 months or older. V_1_, V_2_ and V_3_ are the birth-cohort coverages of MCV1, MCV2 and SIA, b is the birth rate and 𝐅𝐎𝐈 is the force of infection.1b shows the MCV1, MCV2 coverage and timing and target age groups of past SIAs in Kenya. 1c shows the measles case notification data.

We used MCV1 and MCV2 vaccination coverage estimates from a birth-cohort analysis conducted in KHDSS between 2010–2017 [20]. We extrapolated MCV1 coverage estimates for the years that were not included in the birth-cohort analysis from the administrative national MCV1 coverage in Kenya [21] after adjusting for differences based on the comparison for the years we had both datasets (see note A in S1 Text). We relied on the schedule and coverage estimates of historical national SIAs over the course of our study period. These included an SIA in 2002 that offered a single dose of MCV to children younger than 14 years and achieved an estimated 94% coverage followed by SIAs in 2006, 2009 and 2012 in children under 5 years with 90%, 82% and 92% coverage respectively [4]. Additionally, a 2016 measles SIA in children aged 9 months to 14 years achieved a coverage of 95% [22] (Fig 1b).

### Measles case data

Both national and sub-national measles case data rely on hospital admissions. Clinicians report suspected measles cases to disease surveillance coordinators in the county, and laboratory confirmed cases are entered into a central database and reported to WHO [4]. KHDSS measles case notification data was only available from 2014 onwards. We used national measles case data reported to WHO [23] (Fig 1c) as a proxy for cases in KHDSS after identifying matching trends for the years in which both datasets were present (Fig A in S1 Text).

### Model structure

We used a static birth cohort model to track the proportion of children who are either susceptible to measles seroconversion (S) or seroconverted due to natural infection (NI) or due to vaccination with MCV1 (MCV1), MCV2 (MCV2) or SIA (SIA) (Fig 1a). The population is structured by age and calendar year. The age structure is composed of monthly age groups for the first 2 years of age with annual age groups thereafter up to 14 years. We fitted the model to serological data and measles case notification data to estimate the relative contributions of these seroconversion routes to measles immunity in Kilifi.

We assumed that all infants are born with maternal immunity that lasts for 6 months [24] after which they become susceptible and can get infected with a probability governed by the force of infection (FOI). The FOI was assumed to be age-independent but proportional to the year to year change in the reported number of measles cases and thus defined as $FOI=\;\overset{―}{FOI}*C/\;\overset{―}{C}$, where FOI is the annual and $\overset{―}{FOI}$ the average FOI during the study period, and C and $\overset{―}{C}$ the annual and average reported number of measles cases respectively. We assumed that case ascertainment sensitivity remained similar during the study period.

MCV1 vaccination in the model was assumed to be administered at 11 months based on estimates on the timeliness of vaccinations in the KHDSS [20] and elsewhere [25,26]. MCV2 in the model was given at 18 months as there was insufficient evidence of delay in timeliness. SIAs were implemented at the timing of their conduct in Kilifi. We modelled vaccine efficacy as all or nothing whereby a proportion of the vaccinated population seroconverted, and the remainder was considered a vaccine failure and remained susceptible. We did not asses the build-up of immunity in an individual from subsequent doses but rather assumed that individuals who recover from measles infection or seroconvert after vaccination are immune [11].

### Model calibration

The model was initiated in 1996, corresponding to the birth year of the oldest age group in the initial serological survey (2009) and run until 2021. Utilizing monthly timesteps, the model adopted a fixed age of vaccine administration at 11months for MCV1 and 18 months for MCV2. Additionally, it incorporated the schedules and timing of SIAs over the study period to derive modelled seroconversion rates for the age cohorts in 2009 and subsequent surveys. We fitted the model to observed age-and year-specific seroprevalence data by estimating four parameters: the probability of seroconversion following (i) a dose of MCV to children younger than 12months (MCV1 or SIA), (ii) a dose administered to children between 12–18 months old (MCV2 or SIA) and (iii) a dose administered to children older than 18months (SIA) as well as (iv) the average force of infection.

We assumed a beta distribution for the priors of seroconversion of vaccine administered to children younger than 12months with mean of 84% and with a mean of 93% for older children [27]. We used a non-informative prior for $\overset{―}{FOI}$. Posterior distributions for the estimated model parameters were inferred using a Markov chain Monte Carlo (MCMC) approach with adaptive Metropolis-Hastings algorithm to maximise the binomial likelihood of the observed measles serological profile. The Gelman-Rubin statistic was used to evaluate MCMC convergence using a threshold of <1.1 while effective sample size (ESS) calculation was conducted to check for autocorrelation in the MCMC chains. The model was coded in R and the code is available in https://github.com/CarolineNM/Measles-seroconversion.git. Model and likelihood equations are provided in the supplementary material (see note B in S1 Text). The model parameters are described in Table 1.

**Table 1:** Overview of the model parameters and sources for the primary analyses.

Symbol	Definition	Value	References
Ɛ_1_	1-vaccine failure rate of one dose given at <12months	Estimated, prior Beta distributed: 84% (72 - 95%)	[27]
Ɛ_2_	1-vaccine failure rate of one dose given between 12–18months	Estimated, prior Beta distributed: 93% (85 - 97%)	[27]
Ɛ_3_	1-vaccine failure rate of one dose given at >18months	Estimated, prior Beta distributed: 93% (85 - 97%)	Assumed
FOI	Force of infection	$\overset{―}{FOI}*C/\;\overset{―}{C}$	
$\overset{―}{FOI}$	Average force of infection	Estimated, uninformative prior	
C	Annual reported cases of measles	WHO reported cases for Kenya in1996–2021	[12]
$\overset{―}{C}$	Average annual number of reported measles cases	C/ 26	
*V*_*1*_	Annual birth-cohort coverage of MCV1	See Fig B in S1 Text & note B in S1 Text	[20]
V_2_	Annual birth-cohort coverage of MCV2	See Fig B in S1 Text & note B in S1 Text	[20]
V_3_	Coverage of SIA	94%, 2002, 9months-14yrs	[4,22,28]
		90%, 2006, 9months- < 5yrs	
		82%, 2009, 9months- < 5yrs	
		90%, 2012, 9months- < 5yrs	
		95%, 2016, 9months-14years	
	Average age of routine vaccination	MCV1: 9 months+8weeks	[20,25,26]:
		MCV2: 18 months	
	Duration of maternal immunity	6months	[24]

### Model projections (counterfactual scenarios)

We used the fitted model to assess the impact of two of the main challenges in the current vaccination programme: suboptimal MCV1 and MCV2 coverage and suboptimal timeliness of MCV1.

Drawing from the posterior parameter estimates we re-ran the model during the post-MCV2 introduction period with the same FOI and the same timing and coverage of SIAs but

1) assumed that MCV2 coverage had been higher, at half way between the actual coverage and the MCV1 coverage (scenario: “higher MCV2”),
2) assumed that MCV2 coverage had been as high as the observed MCV1 coverage (scenario: “very high MCV2”),
3) assumed that MCV1 coverage had been 10% higher than observed and matched by MCV2 coverage. (scenario: “higher MCV1/2”),
4) the same as scenario 3) but with timely delivery of MCV1 as scheduled at 9 months (scenario: “timely & higher MCV1/2”),
5) assumed timely delivery of MCVI at 9months and MCV1 and MCV2 coverage of 95% (scenario:” ideal MCV1/2”)

### Sensitivity analysis

We conducted a sensitivity analysis to assess the impact of the age cut off of priors of vaccine failure rates on the relative contribution of MCV1 and MCV2 to seroconversion by varying the age limit associated with each dose by 2 months and by 4 months. We also assessed the sensitivity of our findings to timeliness in receipt of MCV1 by varying the age of administration of an MCV1 dose to 9 and 10months. Finally, we evaluated the sensitivity of the seroconversion rates following vaccination to determine if the data significantly updates the priors. This was achieved by assuming non-informative priors, with a mean of 50%, for vaccines administered to both younger and older children.

## Results

### Description of the data and parameter estimates

Overall, 2414 (90%) of 2686 samples tested in this study had measles IgG antibody concentrations indicative of previous exposure either through infection or vaccination. The proportion of children with protective measles antibodies in Kilifi increased from 88% CI: (81–92%) in 2009 to 93% CI: (90–97%) in 2021 (Fig B in S1 Text). In all the surveys, seroprevalence increased early in life, in the months following the scheduled administration of MCV1 with little discernible trend thereafter. MCV1 coverage ranged from 75% to 85% between 2009 and 2021. Notably, it increased between 2012 and 2016 but subsequently declined, remaining below 80% for the remainder of the study period. The number of reported measles cases was higher in the early years and averaged 2000 cases annually between 1996 and 2021 (Fig 1c). The observed peaks in 2011, 2014, 2018 and 2020(Fig B in S1 Text) coincided with declared national measles outbreaks in Kenya.

**Fig 2:**
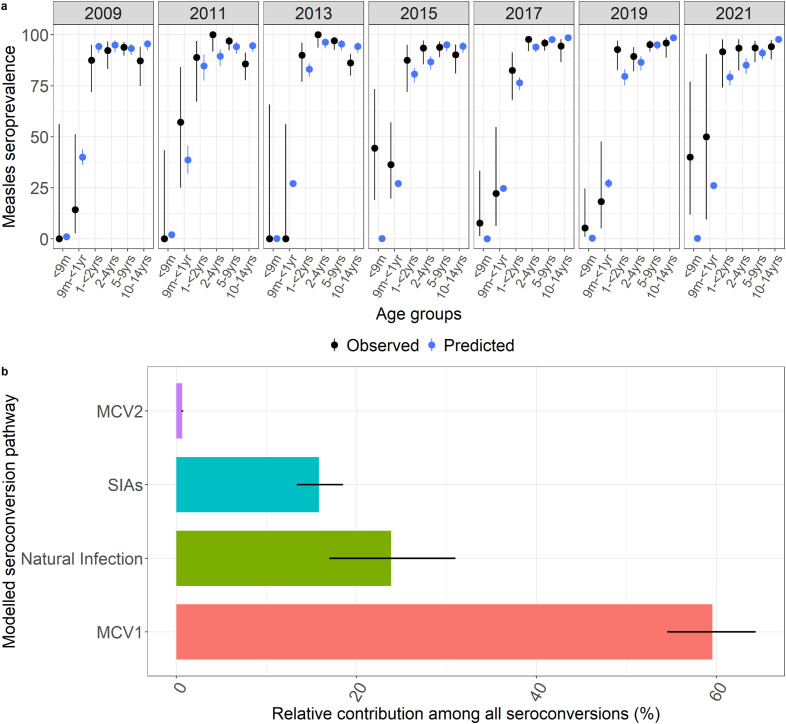
2a shows the age-specific serology data in children grouped into <9m (ineligible for vaccination), 9m- < 1year (eligible for MCV1),1- < 2 years (eligible for MCV2), 2–4 years (eligible for SIA in under 5), 5–9 and 10–14 years (eligible for SIA in under 14). Black is the observed data with 95% confidence intervals. The blue is the estimated seroprevalence sampled from the fitted model with 95% credible interval of the predictive posterior distribution. Model shows a good fit with majority of the predicted seroprevalence falling within the 95% CI of the observed seroprevalence. 2b is the modelled output showing percentage of children that seroconverted either through MCV1, MCV2, SIA or natural infection between 2009 and 2021. Error bars indicate the credible interval of the predictive posterior distribution.

The model was able to fit serological data well (Figs 2a and C in S1 Text). The estimated probability of seroconversion following vaccination was similar in the first two years of life at 93% (CI 89–97) for a dose administered to children less than one year and 93% (CI 86–97) for a dose administered to children between the ages of 12–18 months. The estimated probability of vaccine induced seroconversion in older children was 76% (CI 71–84) (Table 2). The average monthly FOI was estimated to be 6.0% (CI 3.5–9.1%) between 1996 and 2021. After adjusting for the lower case burden in more recent years, this corresponds to an estimated 16% of children in Kilifi being exposed to measles annually between 2009 and 2021 (Table 2 and note C in S1 Text)

**Table 2:** Parameter estimates from the static cohort model. Gelman-Rubin statistic is < 1.1 implying successful convergence of the chains.

Parameters	Definition	Low	Median	Upper	Rhat	Effective Sample size
Ɛ_1_	1-vaccine failure of one dose given at <12months	0.885	0.931	0.966	1	2164
Ɛ_2_	1-vaccine failure of one dose given between 12–18months	0.860	0.930	0.970	1	1884
Ɛ_3_	1-vaccine failure of one dose given at >18months	0.710	0.756	0.849	1	2002
$\overset{―}{FOI}$	Average monthly force of infection	0.035	0.060	0.091	1	2035

### Relative contributions of vaccination to measles seroconversion

The model estimated that administration of MCV1 in Kilifi accounted for 60% (CI 55–64%) of the seroconversion between 2009 and 2021 while natural infection contributed 24% (CI 17–31%) (Fig 2b). The contribution of natural infection declined towards the end of the study period accounting for 12% (CI 8–16%) of seroconversion in 2021 compared to 42% (CI 32–49%) in 2009 (Fig D in S1 Text). SIAs were estimated to have contributed 16% (CI 14–19%) of all seroconversion over the study period with similarly decreasing contribution towards the end of the study period. MCV2 only contributed 1.0% (CI 0.9-1.1%) in the years since its introduction in part due to its low uptake to date (Fig E in S1 Text).

### The potential impact *of* increased routine MCV coverage

**Fig 3:**
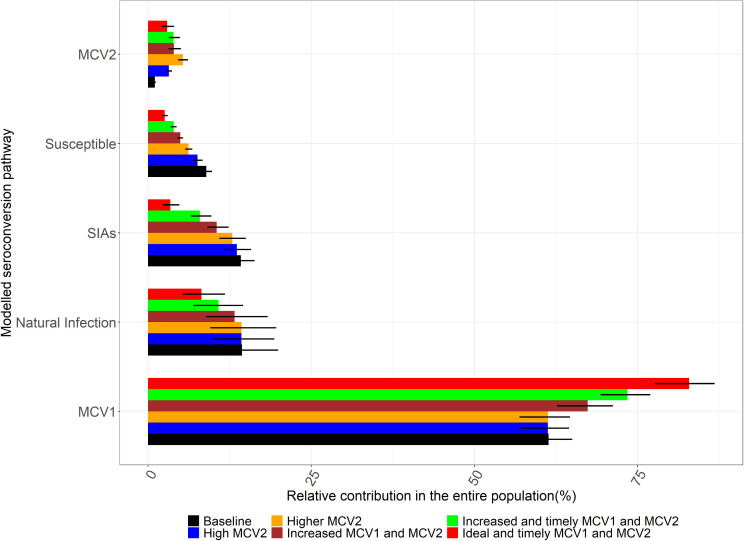
Estimated seroconversion profiles from the projection scenarios on increased MCV1 and MCV2 coverage. In the higher and very high MCV2 scenarios, there is a substantial increase in the relative contribution of MCV2 and a slight decline in the proportion susceptible. Increasing both MCV1 and MCV2 coverages as well as MCV1 timeliness has a considerable impact on the proportion seroconverted from all the pathways.

Predicted measles seroprevalence was higher in all the projection scenarios compared to baseline seroprevalence (Fig F in S1 Text).When MCV2 coverage was increased, the model predicted a decline in residual susceptibility and the contribution of SIAs to seroconversion (Figs 3 and G in S1 Text). The relative contribution of MCV2 in the period since MCV2 introduction increased from 1.0% (CI 0.9-1.1%) currently to 3.2% (CI 2.8-3.7%) and 5.3% (CI 4.6-6.1%) when assuming higher MCV2 and very high MCV2 coverage. This increase corresponded to a decline of the proportion susceptible from 8.9% (CI 8.2-9.8%) currently to 7.6% (CI 7.0-8.3%) and 6.2% (CI 5.7-6.8%) in the two scenarios respectively.

Increased MCV1 and MCV2 coverage was predicted to substantially reduce the contribution of SIAs and natural infection to seroconversion (Fig 3). In addition, increased and timely MCV1 and MCV2 was estimated to reduce seroconversion from natural infection and SIAs even further. A 10% increase in the MCV1 coverage increased the proportion of seroconversion attributed to MCV1 from 61% (CI 57–65%) to 67% (CI 63–71%) and further to 73% (CI 69–77%) if MCV1 is administered at the recommended 9 months of age. With 95% coverage for each of MCV1 and MCV2, 83% (CI 78–87%) of seroconversions were attributable to MCV1 and this reduced the contribution of natural infection to seroconversion to 8% (CI 5–12%) and SIAs to 3% (CI 2–5%).

### Sensitivity analysis

The relative contribution of MCV1 and MCV2 towards seroconversion was not sensitive to alternative assumptions of age limits for the priors of the vaccine failure rates in the model. Increasing the age limits of vaccine failure of a dose from <12months, 12–18months and >18months to vaccine failure of a dose from <16months, 16–22months and >22months resulted in highly similar estimates of the relative contribution of the different programs (Fig H in S1 Text).

Timeliness of MCV1 however had a considerable impact on the modelled seroconversion estimates. Seroconversion attributable to MCV1 increased from 60% (CI 55–64%) to 64% (CI 59–68%) if we assumed that MCV1 was administered at 10months and further to 66% (CI 60–71%) if assumed to be administered at 9 months in the baseline model (Fig I in S1 Text).

The estimated probability of seroconversion following vaccination was not sensitive to alternative assumptions of priors of the vaccine failure rates in the model. Similar to the main model, the estimated probability of vaccine induced seroconversion in the first two years of life was 93% (CI 89–97) for a dose administered to children less than one year and 93% (CI 87–97) for a dose administered to children between the ages of 12–18 months. The estimated probability of vaccine induced seroconversion in older children was 76% (CI 69–84).

## Discussion

Availability of historical serological data from a series of cross-sectional surveys over a period of 12 years in a Kenyan population enabled us to estimate the relative contribution of natural infection and the different immunisation programs to measles seroconversion. We found that MCV1 accounted for more than 50% of the seroconversions in the study period. Natural infection and SIAs led to 24% and 16% of all seroconversions respectively, illustrating the reliance of the current RI programme on SIA and the size of the immunity gap allowing measles circulation. We find that increasing the coverage of MCV2 and particularly that of MCV1 and most importantly improving the timely administration of MCV1 has the potential to substantially reduce the need for SIAs and reduce the burden of measles in Kilifi.

We based our analysis on a static cohort model of measles immunity fitted to observed serology data and incorporating measles case-notification data and records on timing, schedule, and coverage of RIs and SIAs in the country. Coverage and timing of the different vaccine doses allowed us to estimate the increasing rate of seroconversion from vaccination in the different age groups while increasing rate of seroconversion as a result of natural infection was estimated by a probability governed by FOI which was assumed to be proportional to the year to year change in the reported number of measles cases. We did not consider the additional vaccine protection as a result of a second dose in already immunised individuals but rather, and in line with the rationale of WHO to recommend a second dose, assumed that the main benefit of MCV2 would be to provide protection to those unprotected from MCV1 either due to vaccine failure or because of missed vaccination [11].

Our main findings and projection scenarios align with the WHO recommendations around measles vaccination strategies [11]. MCV1 remains the most crucial of the immunisation opportunities in the current program for measles control, accounting for by far the greatest contribution of the seroconversions if given early in life and at high coverage. These findings are in line with similar studies where contribution of MCV1 to seroconversion was estimated to be as high as 90% in some countries [28]. We estimate that if MCV1 coverage had been 10% higher during the study period residual susceptibility to infection would have been about 50% lower and at the same time would have reduced the need for reliance on SIA, highlighting the potential of seemingly small improvements in coverage to reduce the burden from measles and reduce the costs associated with SIAs. A possible way to increasing MCV1 uptake could be the roll out of the new RTS,S malaria vaccine which is scheduled to be given to infants aged between 5–17 months in a four dose schedule [29]. This follows from previous findings that have shown strengthening of service delivery in already existing routine vaccine programs as a result of new vaccine introduction [30].

The recommended age of MCV1 receipt is 9 months in a high transmission setting. In the model, delivery of MCV1 at 9 months resulted in 7% more seroconversions compared to MCV1 delivered at 11months with a similar coverage suggesting that efforts on ensuring optimal timing needs to be emphasized as even one-month delay may have substantial impact on the risk for natural infection [20]. We did not assess the effect of administering MCV1 to infants younger than 9 months. Although this early vaccination has been proposed in high risk settings, there is a moderate evidence showing that it might negatively impact seroconversion to subsequent measles vaccine doses [31].

In 2019, WHO initial recommendation was for introduction of MCV2 when countries achieved 80% MCV1 coverage but this was revised in 2017 to a recommendation to introduce MCV2 regardless of MCV1 coverage. The goal was to target unvaccinated children missed by earlier doses, particularly in LMICs where SIAs are not often implemented on schedule [11]. We show that, so far, this 2^nd^ dose has led to 1% to the total seroconversions since it was first introduced, in part due to the low vaccination coverage However, there has been a gradual rise in the annual relative contribution to seroconversion, increasing from 0.2% in 2015 to 3% in 2021 primarily due to the gradual but consistent improvement in coverage over the years. Similarly low MCV2 coverage has been reported across Africa and has been attributed to several factors including the knowledge, perception and attitudes towards the vaccination at both the individual and community level [32]. The timing of the dose which was arrived at based on programmatic considerations [11] is also a factor as it does not correspond with the schedule of the other routine immunisation vaccines at the moment. Similar to MCV2, the roll-out of RTS,S malaria vaccine may provide an opportunity to increase awareness and coverage of MCV2 [29]. However, we show that increasing MCV2 coverage, while important to minimise the immunity gap from MCV1 failures, is likely to have much less impact than any increase in MCV1 coverage.

Frequent SIAs are recommended to close immunity gaps especially in communities who are hard to reach with RI programmes. However, SIAs are expensive and their impact is often diminished by delays or disruptions especially in LMICs as well as the inability to reach zero dose children [33]. In total five SIAs all with high coverage were conducted in the 12-year study period and were estimated to have contributed about 14% of seroconversions. This impact of SIAs while still crucial to reduce the immunity gaps left by insufficient RI coverage would likely be smaller if SIAs are delivered to children who are already vaccinated. This has been shown to be one of the main challenges with measles SIAs [33] and among the reasons for recommendations by WHO to phase out SIAs once countries achieve a 95% coverage of both doses. Our findings concur with the recommendation as increasing RI coverage to 95% in the model would reduce the contribution of SIAs to total seroconversions to only 3%.

A key strength of our study was the availability of good quality historical serology data spanning over a decade which provided excellent means of directly estimating levels of population protection. The KHDSS population registry was crucial in informing assessment of vaccination coverage [20] while mathematical modelling allowed us to combine these different pieces of evidence to make meaningful conclusions on the current immunity profile in children in Kilifi.

Our study had a few limitations. First, we used a static cohort model in our projections which is bound to reduce the impact of vaccination as we do not account for the indirect herd effects of vaccination; these will particularly underestimate the ability to control measles circulation at high vaccine coverage. As a result, it may underestimate the decline in natural infection–driven seroconversions. However, other sources of bias such as assumptions about vaccine coverage, case reporting, and model structure could influence estimates in either direction.. We used nationwide measles case data in our projections as we did not have estimates from KHDSS for all years of study. However, there were similar trends of measles cases in KHDSS and nationwide for the years in which both datasets were present and there was also no evidence suggesting that measles case finding changed during the study period. Our analysis also relied on rural data, restricting the generalisability to diverse contexts. While the results represent rural measles-endemic areas well, variations may occur in urban settings primarily due to differences in vaccination coverage and measles susceptibility profiles in urban and rural settings.

The MCV1 and MCV2 coverage estimates used in the analysis were informed by a birth-cohort analysis and administrative nationally reported coverage estimates. Given the limitations associated with the methods of collecting this data, it is likely that the estimates could either be an overestimate or underestimate. If both MCV1 and MCV2 were overestimated, this would likely mean that we underestimated the impact of SIA in our model. Conversely, if both MCV1 and MCV2 coverages were underestimated, this would imply that we overestimated the relative contribution of SIAs to the total seroconversions. Additionally,in the later years of the study period (2015–2021), the model underestimated seroprevalence in some younger age groups, particularly in 2021. This could reflect local transmission not captured in national case data, earlier-than-assumed MCV1 administration age or prolonged maternal antibody protection. These factors may explain the observed high seroprevalence in infants and the seeming incongruity between high seroprevalence in the youngest and lower predicted immunity in slightly older age groups.

Finally, we did not incorporate build-up of immunity from subsequent doses in individuals. Instead, our assumption was that immunity from a single dose was lifelong and that the administration of MCV1, MCV2, and SIAs is uncorrelated. In reality, health-seeking behavior could lead to correlations, as children receiving MCV1 are likely to receive MCV2 and participate in SIAs, an observation reinforced by the fact that SIAs have not significantly decreased the number of children with zero vaccine doses [33]. These structural assumptions likely contributed to the lower estimated seroconversion rate for first doses given after 18 months. Many children in this subgroup may have already seroconverted through earlier infection or vaccination and were therefore no longer eligible to seroconvert in the model. As a result, the model may have attributed fewer seroconversions to these later doses, leading to an underestimated vaccine effect. Although this assumption simplified our cohort model and is not expected to marginally change the outcome, it is likely that we slightly overestimated the impact of MCV1 and underestimated the impact of MCV2.

In conclusion, a combination of serological, vaccine coverage and measles surveillance data and mathematical modelling allowed us to assess the impact of the current measles prevention programme in Kilifi by deciphering the relative contributions of MCV1, MCV2, SIAs and natural infection to measles seroconversions. We showed that a slight increase in routine vaccination coverage and timeliness, especially of MCV1, can result in a substantial decline on the reliance on SIAs as well as the prevention of natural infection. Particularly the roll out of RTS, S may provide an opportunity to increase routine Measles vaccination coverage to improve measles control and reduce costs associated with frequent SIAs.

## Supporting information

S1 Text Note A in S1 Text. Extrapolation of vaccination coverage estimates. **Note B in S1 Text.** Model equations. **Note C in S1 Text.** Calculation of annual FOI. **Fig A in S1 Text**.Annual relative Measles cases. **Fig B in S1 Text**: MCV1 coverage, measles cases and seroprevalence estimates between 2009 and 2021. **Fig C in S1 Text**. Convergence chains. **Fig D in S1 Text**. Estimated age-specific measles immunity profiles. **Fig E in S1 Text**. Modelled seroconversion pathways between 2015-2021. **Fig F in S1 Text**. Predicted measles seroprevalence for the counterfactual scenarios. **Fig G in S1 Text**: Estimated relative contribution of the different programs from the projection scenarios on increased MCV1 and MCV2 coverage over the entire period (2009-2021). **Fig H in S1 Text**. Impact of age cut-off priors of the vaccine failure on the relative contribution of the different programs to seroconversion.
(DOCX)

## Notes

https://github.com/CarolineNM/Measles-seroconversion.git
