# Supplementary material for: Modelling the relative contribution of infection, routine vaccination and supplementary immunisation activities to measles seroconversion in Kenyan Children
Source: PLoS Comput Biol. 2025 Sep 22;21(9):e1013531. doi: 10.1371/journal.pcbi.1013531 (PMC12469164; doi:10.1371/journal.pcbi.1013531)
Supplement: S1 Text — Note A in S1 Text. Extrapolation of vaccination coverage estimates. Note B in S1 Text. Model equations. Note C in S1 Text. Calculation of annual FOI. Fig A in S1 Text.Annual relative Measles cases. Fig B in S1 Text: MCV1 coverage, measles cases and seroprevalence estimates between 2009 and 2021. Fig C in S1 Text. Convergence chains. Fig D in S1 Text. Estimated age-specific measles immunity profiles. Fig E in S1 Text. Modelled seroconversion pathways between 2015-2021. Fig F in S1 Text. Predicted measles seroprevalence for the counterfactual scenarios. Fig G in S1 Text: Estimated relative contribution of the different programs from the projection scenarios on increased MCV1 and MCV2 coverage over the entire period (2009-2021). Fig H in S1 Text. Impact of age cut-off priors of the vaccine failure on the relative contribution of the different programs to seroconversion. (DOCX) [file pcbi.1013531.s001.docx]

**Supplementary materials for Modelling the Relative Contribution of Infection, Routine Vaccination and Supplementary Immunisation Activities to Measles Seroconversion in Kenyan Children**

**Authors**

Caroline Mburu ^1,2*^, John Ojal ^1,2^, Rose Selim ^1^, Rose Ombati ^1^, Donald Akech ^1^, Boniface Karia ^1^, James Tuju ^1^, Antipa Sigilai ^1^, Gaby Smits ^3^, Pieter van Gageldonk ^3^, Fiona van der Klis ^3^, Eunice Kagucia ^1^, Anthony Scott ^1,2^, Ifedayo Adetifa ^1,2^, Stefan Flasche ^2,4^

affiliations:

^1^ KEMRI-Wellcome Trust Research Programme, Kilifi, Kenya

^2^ Department of Infectious Diseases Epidemiology, London School of Hygiene and Tropical Medicine, London, United Kingdom

^3^ Department of Immunosurveillance, Centre for Infectious Diseases Control, National Institute of Public Health and the Environment (RIVM), The Netherlands

^4^ Charite Centre for global Health, Charite-Universitaetsmedizin, Berlin, Germany

^*^ [mburucaroline@gmail.com](mailto:mburucaroline@gmail.com)

Key words: Measles immunity, Serology data, Vaccination Coverage,

**Note A. Extrapolation of vaccination coverage estimates**

Estimates for MCV1 and MCV2 vaccination coverage estimates for birth-cohort analysis in KHDSS were only available between 2010 and 2017[1]. To extrapolate birth-cohort vaccination coverage in KHDSS from administrative national MCV1 coverage [2] beyond this time frame, we first compared the trends and estimates for the time period in which both datasets were present and observed matching trends. On average, administrative coverage estimates were 11% higher and 21% higher than MCV1 and MCV2 KHDSS coverage estimates. We then adjusted the coverage in the rest of the modelled time frame by subtracting this average difference.

**Note B. Model equations**

The static birth cohort model developed to track proportion of children who are either susceptible to measles seroconversion (S) or seroconverted due to natural infection (NI) or due to vaccination with MCV1 (MCV1), MCV2 (MCV2) or SIA (SIA) is described in detail in the main text. Model parameters are also summarized in table 1 of the main text. In the model, children in each age group are divided into 5 compartments. Transitions for each birth cohort is described by a system of ordinary differential equations which incorporates both aging and vaccination coverage.

Children are divided into five mutually exclusive compartments:

S(a, t): Susceptible individuals who have not yet seroconverted

NI(a, t): Seroconverted due to natural infection

MCV1(a, t): Seroconverted due to MCV1

MCV2(a, t): Seroconverted due to MCV2

SIA(a, t): Seroconverted due to SIA

where *a* denotes age group and *t* denotes time. The total number of individuals in each age group is given by:

N(a, t) = S(a, t) + NI(a, t) + MCV1(a, t) + MCV2(a, t) + SIA(a, t)

**Transition Equations**

Children age into the next compartment monthly. Transitions from susceptible to seroconverted states depend on exposure to infection and vaccination.

1. Susceptible:

S(a, t+1) = S(a-1, t)× (1 - P_NI(a-1, t) - P_MCV1(a-1, t) - P_MCV2(a-1, t) - P_SIA(a-1, t))

2. Natural Infection:

NI(a, t+1) = NI(a-1, t) + S(a-1, t) × P_NI(a-1, t)

3. MCV1 seroconversion

MCV1(a, t+1) = MCV1(a-1, t) + S(a-1, t) × P_MCV1(a-1, t)

4. MCV2 seroconversion

MCV2(a, t+1) = MCV2(a-1, t) + S(a-1, t) × P_MCV2(a-1, t)

5. SIA seroconversion

SIA(a, t+1) = SIA(a-1, t) + S(a-1, t) × P_SIA(a-1, t)

**Probability of seroconversion**

Each P_xx(a,t) is calculated based on age-specific coverage and vaccine effectiveness. If vaccination (MCV1,MCV2,SIA) is scheduled for a specific age *a* only, then P_xx(a,t) is non-zero only at that age.

P_NI(a,t) = $\overline{FOI}*C/ \overline{C}$ FOI is assumed age-independent, scaled to reported case data

P_MCV1(a,t) = ε_1 × V1(a,t)

P_MCV2(a,t) = ε_2 × V2(a,t)

P_SIA(a,t) = ε_i × V3(a,t), where ε_i depends on age group:

ε_1 for age <12 months

ε_2 for 12–18 months

ε_3 for >18 months

V1(a,t), V2(a,t), V3(a,t): age-specific vaccine coverage at time t

**Assumptions**

1. Transitions are only allowed from the susceptible compartment.
2. Seroconversion is permanent and individuals do not move between seroconverted compartments.
3. Vaccination uptake is assumed independent across doses.
4. Vaccine efficacy is modeled as all-or-nothing.
5. SIAs are implemented at fixed times and age targets.

**Notes**

While the model assumes independence in uptake, we acknowledge that in real-world settings, MCV2 and SIAs are more likely to be received by children who already received MCV1. These correlations are discussed in the main manuscript as a limitation of the current structure.

**Note C. Calculation of annual FOI**

To calculate the proportion annually exposed to measles between 2009 and 2021, we derived the annual force of infection (FOI) from the average monthly FOI, denoted as $\overline{FOI}$.

The $\overline{FOI}$ estimated by the model over the full time series (1996–2021) was relatively high ($\overline{FOI}$= 0.06), largely due to elevated case numbers in the earlier years. To obtain an average monthly $\overline{FOI}$ specific to the period 2009–2021, we adjusted the model-derived $\overline{FOI}$ by scaling it according to the relative number of cases during that period.

Adjusted $\overline{FOI}=\overline{FOI}*\frac{C_{2009-2021}}{C_{1996-2021}}$

This yielded an adjusted average monthly FOI of approximately 0.014 (or 1.4%) for 2009-2021.

To convert the monthly FOI to an annual probability of exposure, we used the following transformation:

The monthly probability of not being exposed is (1 − $\overline{FOI}$).

The annual probability of not being exposed is (1 − $\overline{FOI}$)^12.

Therefore, the annual probability of being exposed is 1−(1−$\overline{FOI}$)^12^

Substituting  $\overline{FOI}$= 0.014 yields an annual FOI of approximately 0.16 (or 16%), as reported in the manuscript


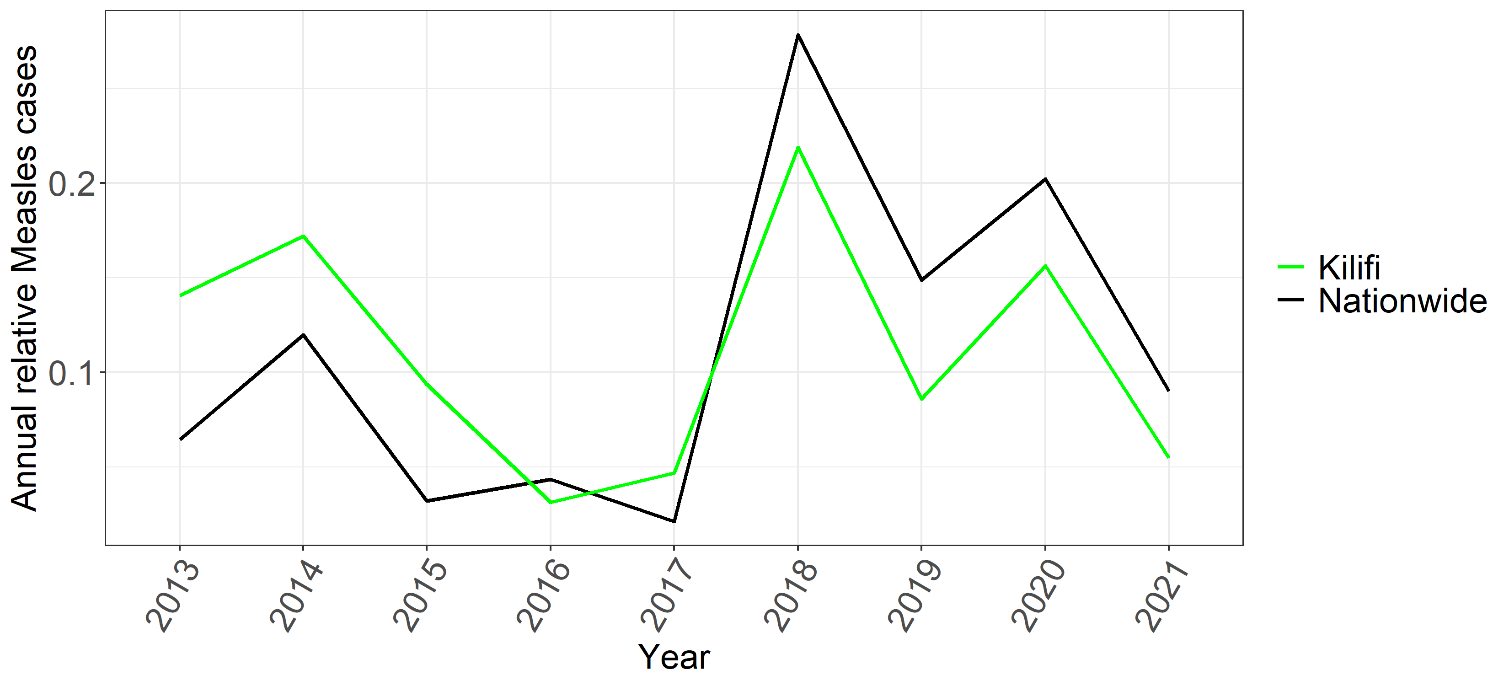


**Fig A**: Comparison of the relative reported nationwide wide cases per year from WHO and the relative reported Measles cases from KHDSS surveillance for the years in which both datasets were present. Annual relative cases were calculated by dividing notified measles cases per year by the total measles cases in the entire period.


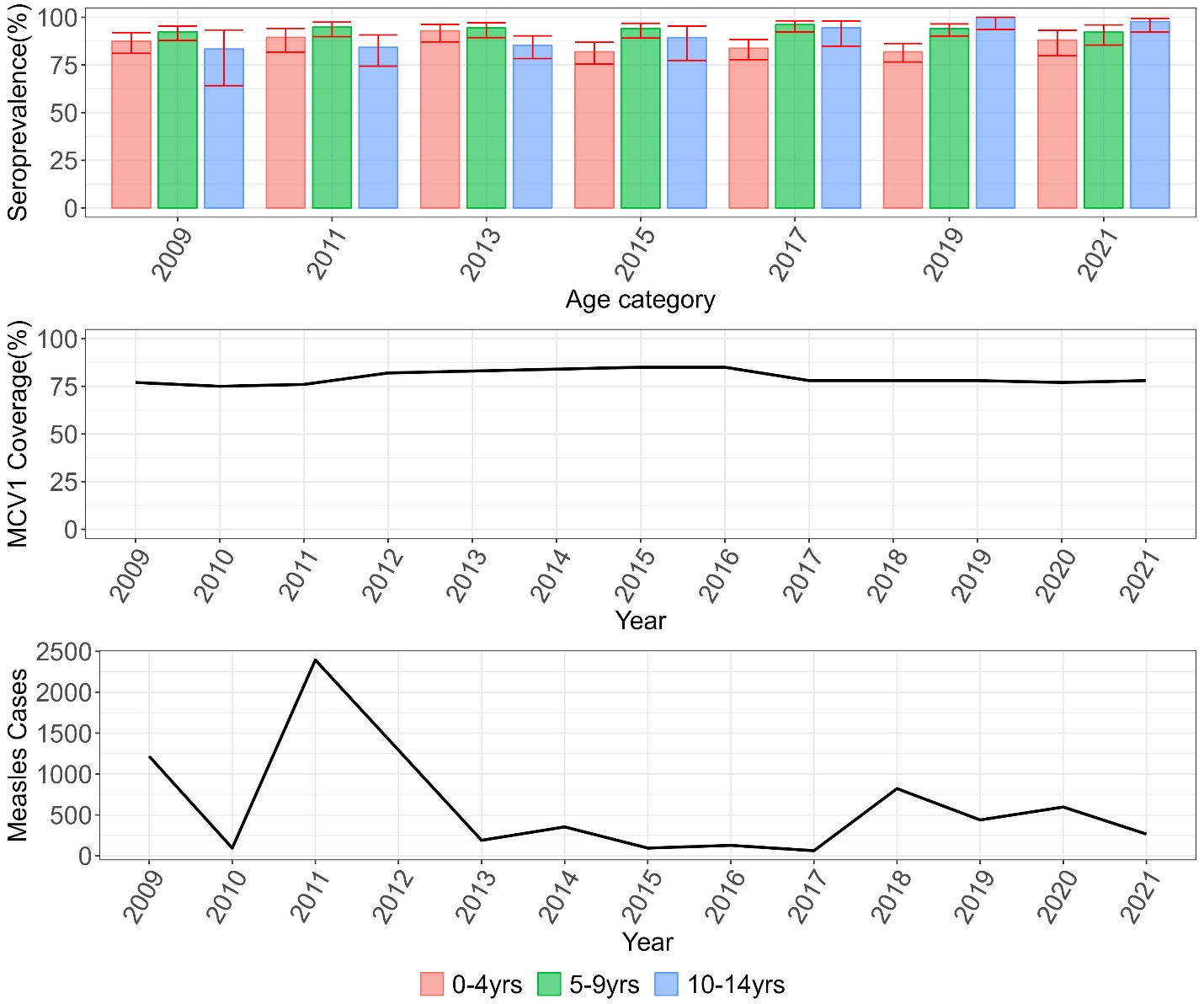


**Fig B**: MCV1 coverage, measles cases and seroprevalence estimates between 2009 and 2021


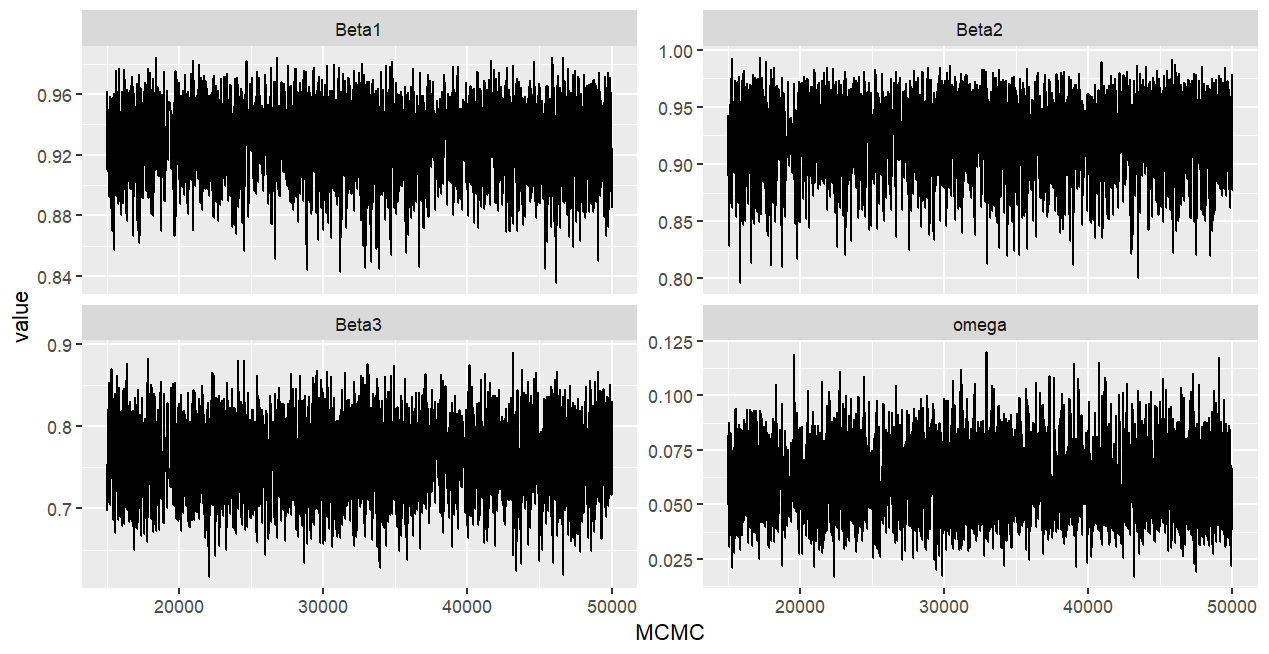


**Fig C**. Convergence chains


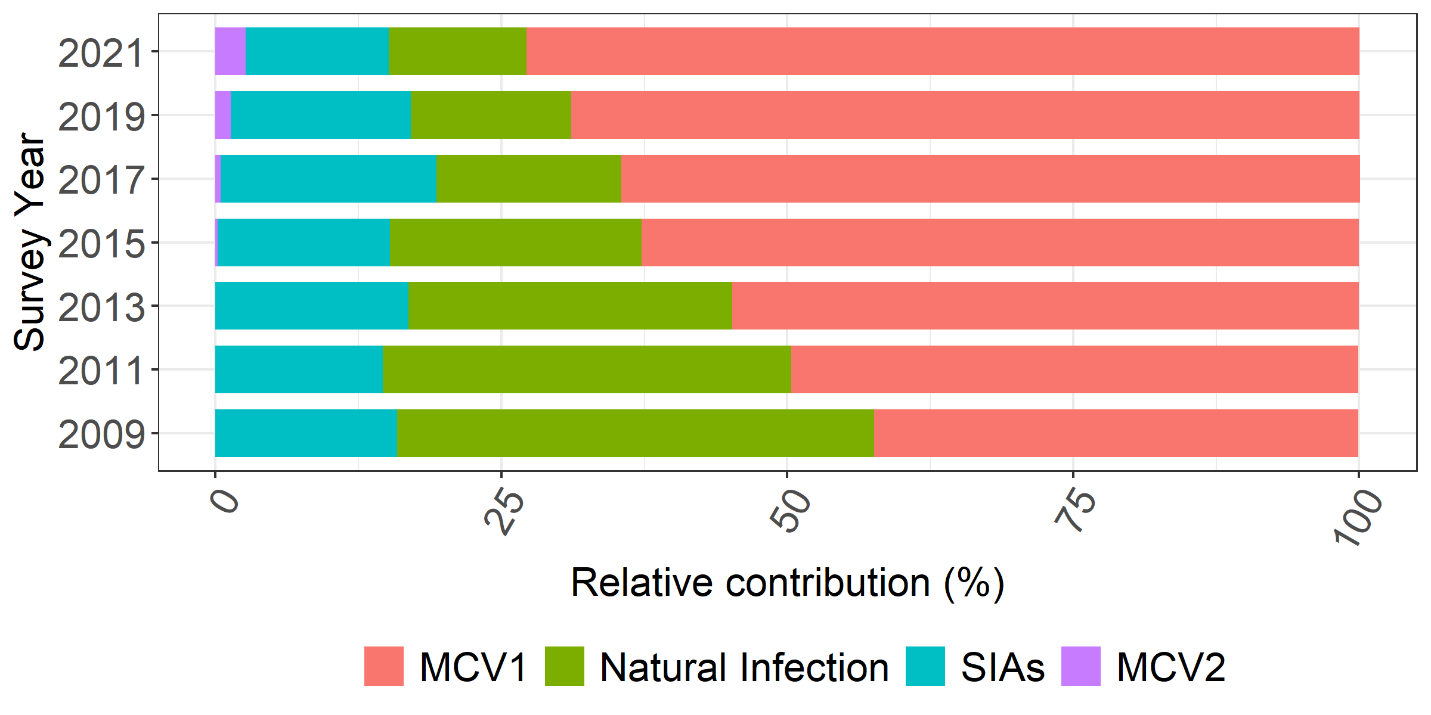


**Fig D**. Estimated age-specific measles immunity profiles. The figure shows percentage of children that seroconverted either through MCV1, MCV2, SIA or natural infection in each year.


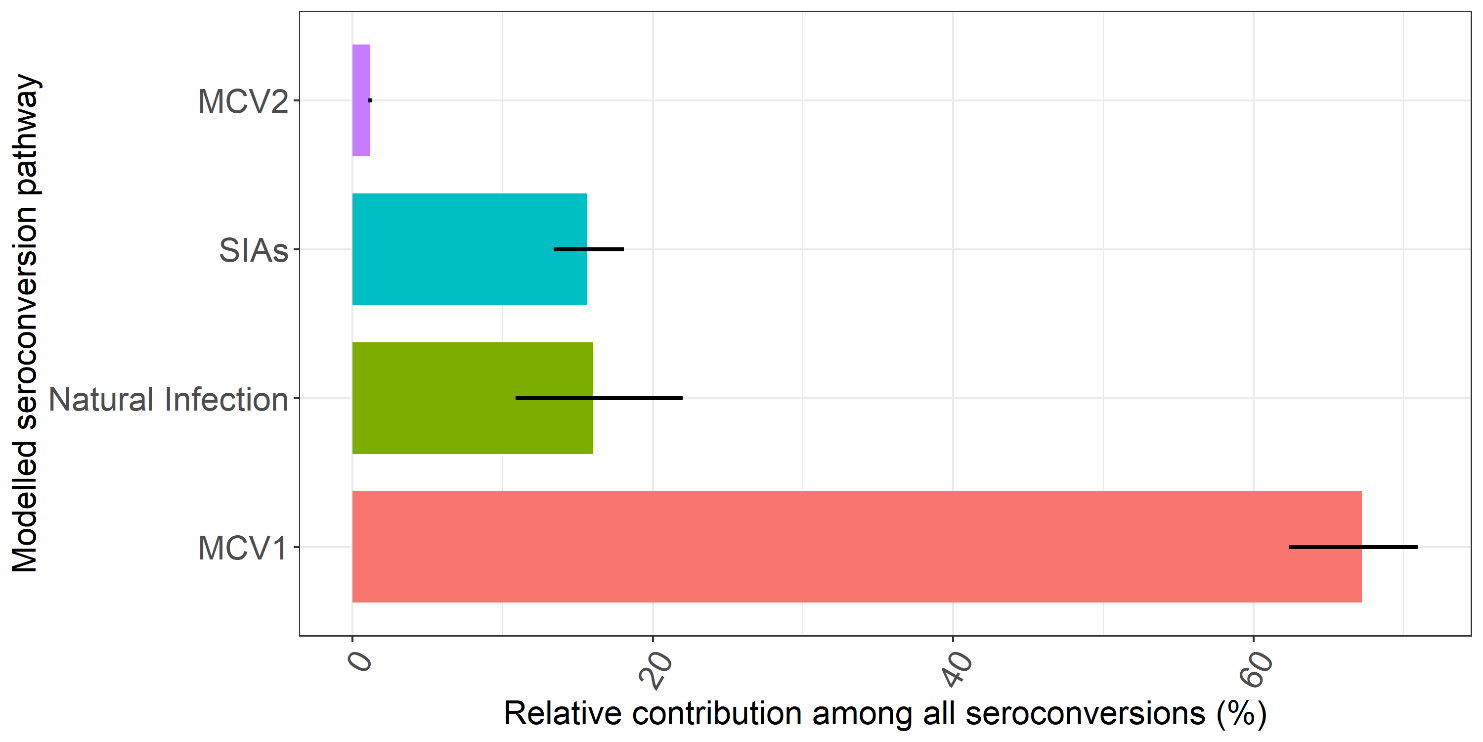


**Fig E**. Percentage of children that seroconverted either through MCV1, MCV2, SIA or natural infection after MCV2 introduction (between 2015 and 2021). Error bars indicate the credible interval of the predictive posterior distribution.

.


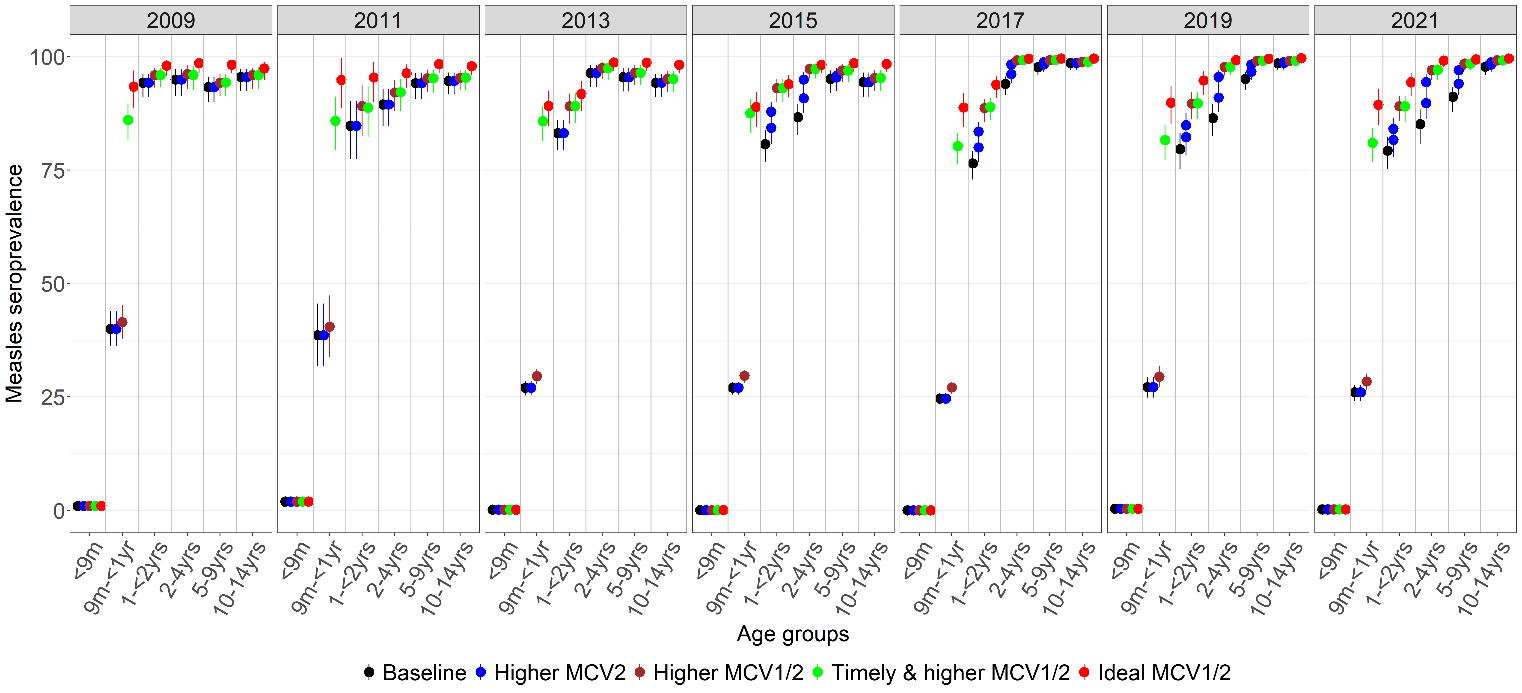


**Fig F**. Predicted measles seroprevalence sampled from the fitted model with 95% credible interval of the predictive posterior distribution


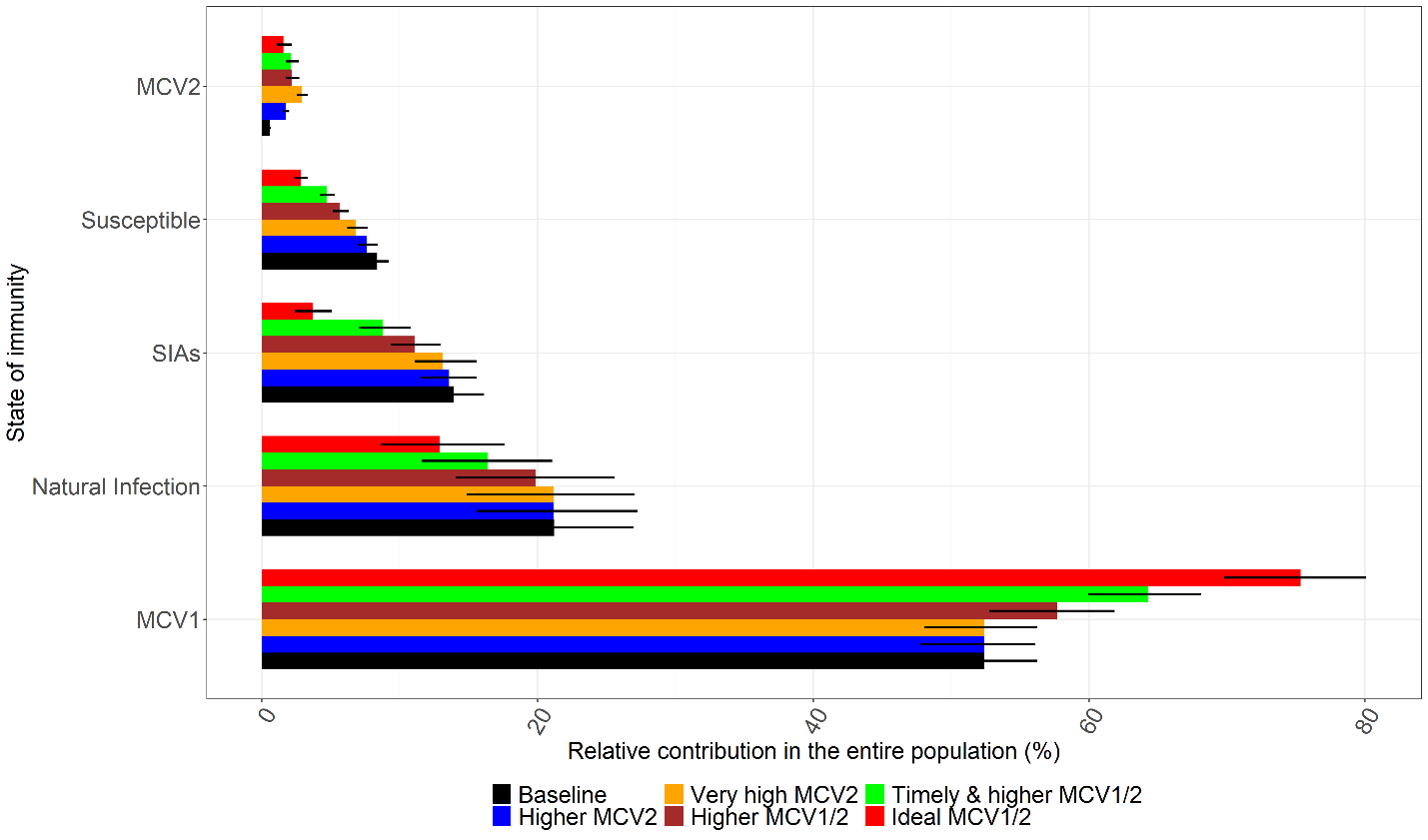


**Fig G**: Estimated relative contribution of the different programs from the projection scenarios on increased MCV1 and MCV2 coverage over the entire period (2009-2021).


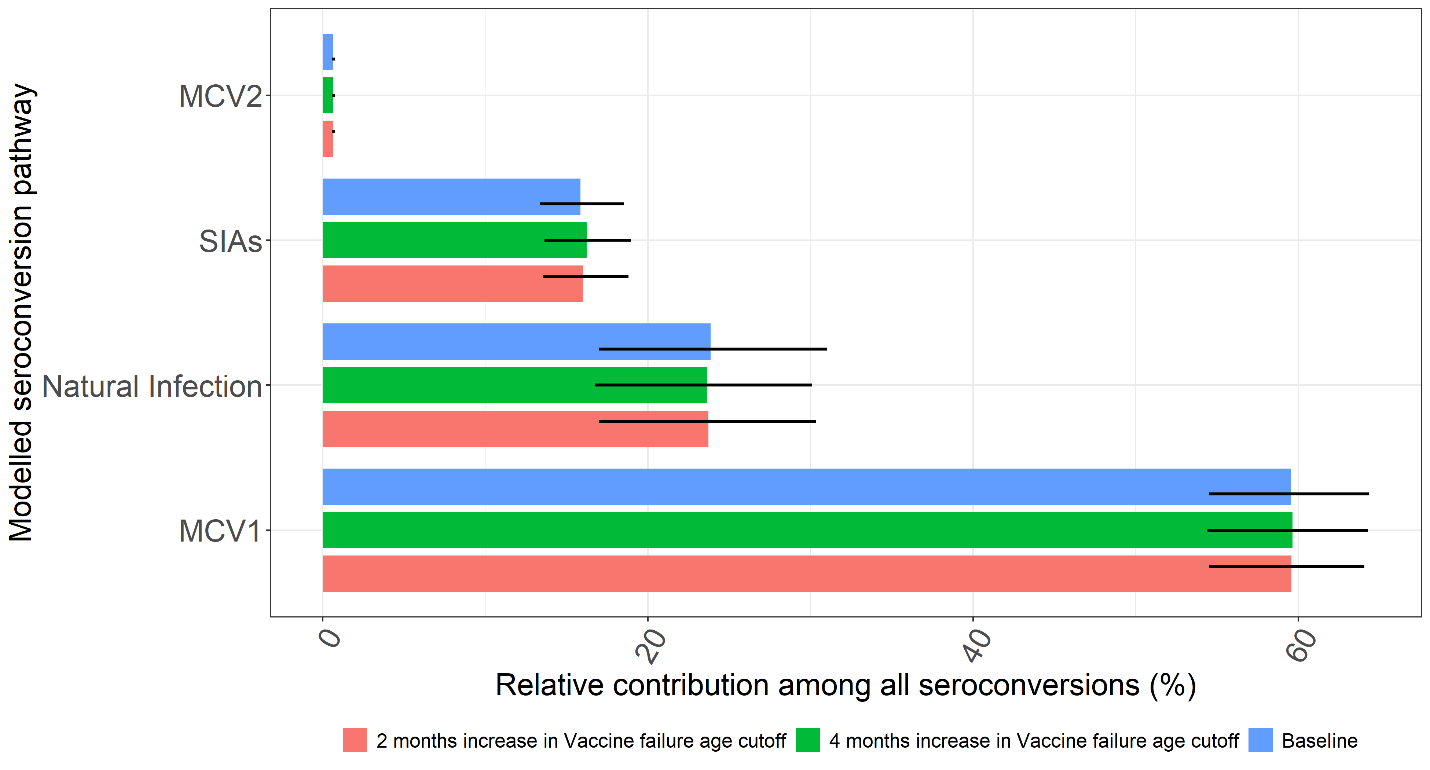


**Fig H.** Impact of age cut off of priors of the vaccine failure on the relative contribution of the different programs to seroconversion


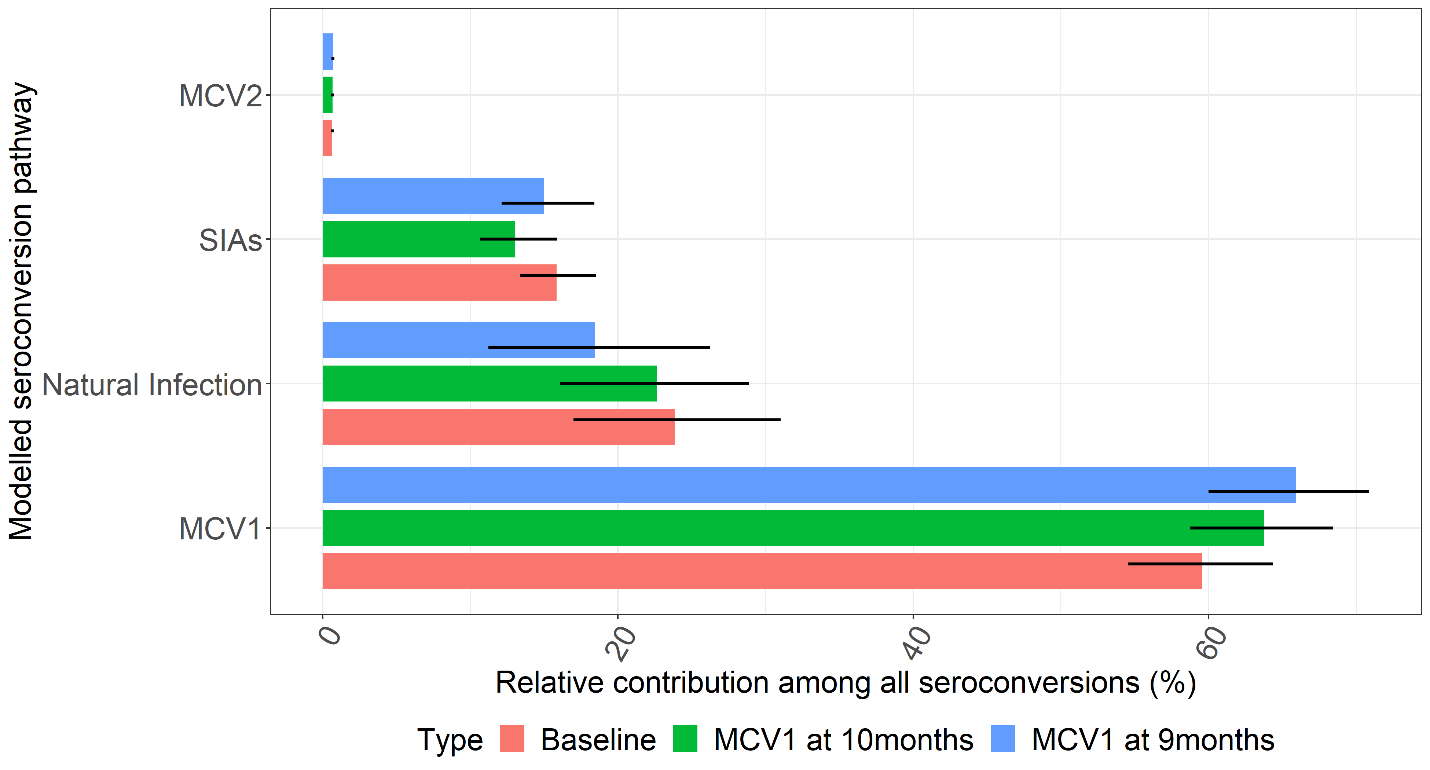


**Fig I**. Impact of timeliness of MCV1 on the relative contribution of the different programs to seroconversion

1. Adetifa, I.M., et al., *Coverage and timeliness of vaccination and the validity of routine estimates: Insights from a vaccine registry in Kenya.* Vaccine, 2018. **36**(52): p. 7965-7974.

2. WHO, *WHO and UNICEF estimates of immunization coverage* [*https://www.who.int/publications/m/item/immunization-kenya-2023-country-profile*](https://www.who.int/publications/m/item/immunization-kenya-2023-country-profile)*.* 2019.
